# Altered white matter microstructure underlies listening difficulties in children suspected of auditory processing disorders: a DTI study

**DOI:** 10.1002/brb3.237

**Published:** 2014-05-29

**Authors:** Rola Farah, Vincent J Schmithorst, Robert W Keith, Scott K Holland

**Affiliations:** 1 Communication Sciences Research Center, Cincinnati Children's Hospital Medical CenterCincinnati, Ohio; 2 Department of Communication Sciences and Disorders, College of Allied Health Sciences, University of CincinnatiCincinnati, Ohio; 3 Department of Radiology, Children's Hospital of Pittsburgh of UPMCPittsburgh, Pennsylvania; 4 Pediatric Neuroimaging Research Consortium, Department of Radiology, Cincinnati Children's Hospital Medical CenterCincinnati, Ohio

**Keywords:** Attention, auditory processing disorder, dichotic listening, diffusion tensor imaging, listening difficulties

## Abstract

**Introduction:**

The purpose of the present study was to identify biomarkers of listening difficulties by investigating white matter microstructure in children suspected of auditory processing disorder (APD) using diffusion tensor imaging (DTI). Behavioral studies have suggested that impaired cognitive and/or attention abilities rather than a pure sensory processing deficit underlie listening difficulties and auditory processing disorder (APD) in children. However, the neural signature of listening difficulties has not been investigated.

**Methods:**

Twelve children with listening difficulties and atypical left ear advantage (LEA) in dichotic listening and twelve age- and gender-matched typically developing children with typical right ear advantage (REA) were tested. Using voxel-based analysis, fractional anisotropy (FA), and mean, axial and radial diffusivity (MD, AD, RD) maps were computed and contrasted between the groups.

**Results:**

Listening difficulties were associated with altered white matter microstructure, reflected by decreased FA in frontal multifocal white matter regions centered in prefrontal cortex bilaterally and left anterior cingulate. Increased RD and decreased AD accounted for the decreased FA, suggesting delayed myelination in frontal white matter tracts and disrupted fiber organization in the LEA group. Furthermore, listening difficulties were associated with increased MD (with increase in both RD and AD) in the posterior limb of the internal capsule (sublenticular part) at the auditory radiations where auditory input is transmitted between the thalamus and the auditory cortex.

**Conclusions:**

Our results provide direct evidence that listening difficulties in children are associated with altered white matter microstructure and that both sensory and supramodal deficits underlie the differences between the groups.

## Introduction

Auditory processing disorder (APD) is a highly heterogeneous, neurodevelopmental disorder defined as a deficiency in the neural processing of auditory stimuli in the central auditory nervous system (CANS) in the presence of normal peripheral hearing (ASHA 2005; BSA 2011). Individuals suspected of APD typically present with listening difficulties and normal audiograms. However, they show abnormal performance on both speech and nonspeech tests of listening. Their listening complaints and symptoms overlap those of other neurodevelopmental disorders (e.g., specific language impairment, attention deficit/hyperactivity disorder, dyslexia; Sharma et al. 2009; Ferguson et al. 2011). In an attempt to maintain the specificity of the APD construct, professional associations (e.g., American Speech Language Hearing Association 2005) excluded deficits in higher order cognition as the underlying cause for APD. However, recent evidence supports the contribution of higher order cognitive abilities to listening difficulties (Moore et al. 2010, 2013). Furthermore, behavioral tests most frequently utilized in the diagnosis of APD have been repeatedly criticized for relying on higher order processing constructs, including memory, language, and attention (Cacace and McFarland 2005, 2013; Moore 2006; Moore et al. 2010). A recent diffusion tensor imaging (DTI) study investigating the neural correlates of several behavioral tests used in the diagnosis of APD (Schmithorst et al. 2011), corroborated this concern by demonstrating that test performance correlated with independent white matter integrity in regions subserving higher order processing constructs. These findings cast doubt on the interpretation of abnormal behavioral task performance as indicative of pure sensory APD, since supramodal neural deficits may alternatively account for deficient performance.

Auditory processing disorder diagnosis relies mostly on patient's symptoms and the results of a behavioral test battery. Dichotic listening tests (DLTs) are frequently chosen as a central component in APD diagnosis to investigate hemispheric asymmetry, language lateralization, central auditory pathway maturation, and auditory attention (Bryden et al. 1983; Bryden 1988; O'Leary 2002; Hugdahl 2003; Keith and Anderson 2007; Takio et al. 2009; Musiek and Weihing 2011). The difficulty of DLTs challenges the auditory system and other higher order systems and can reveal deficits in auditory processing that might go undetected otherwise (Jerger 2006).

In a DLT, different auditory stimuli are presented simultaneously to each ear and the listener is instructed to repeat what was heard. For speech-related stimuli, a finding of right ear advantage (REA; Hugdahl et al. 2003) is typical, reflecting that most individuals report more accurate stimuli presented to their right ear compared to their left ear in the “free-recall” mode (“report both stimuli in any order”). An atypical left ear advantage (LEA; more accurate recall from the left ear) for speech or speech-related stimuli is considered an atypical finding, interpreted as denoting mixed/right-hemisphere language dominance, or an indication for APD (Keith 1984; Zatorre 1989; American Academy of Audiology 2010).

No ear advantage (NEA) or an LEA has been demonstrated in about 20% of the right-handed population (Bryden 1988), and in typically achieving school-aged children (Moncrieff 2011). In contrast, only an estimated 1–5% of right-handed individuals have right-hemisphere lateralization for language processing (Loring et al. 1990; Knecht et al. 2000). Furthermore, the interpretation of LEA as an indication of APD in the presence of listening complaints has not been validated on neurologically intact children. A subgroup of children suspected of APD exhibit an atypical LEA for speech-related stimuli in free-recall DLT; left ear recall outperforms right ear recall. Their associated listening difficulties might indicate a possible neuropathology, not confirmed by behavioral testing. A recent neuroimaging study has been conducted in this group to explicate the neural bases of their listening complaints. Schmithorst et al. (2013) used machine learning techniques on functional MRI (fMRI) and DTI data to predict whether an individual will show an REA or LEA during dichotic testing. The results revealed that LEA for speech-related stimuli was predicted by both sensory and attention deficits. Thus, a LEA finding cannot be taken as a unique indicator of sensory processing deficit; attention deficits can equally account for a LEA.

In this study, we used traditional analysis of scalar DTI measures in conjunction with the white matter atlas to investigate white matter integrity underlying listening difficulties in the same sample of children. DTI is a powerful magnetic resonance imaging technique for examining white matter microstructure, in vivo, by estimating diffusion of water molecules along axonal pathways (Le Bihan et al. 2001). By sensitizing the MR signal to the magnitude and directionality of water movement on a microscopic level, water diffusivity along the three principle diffusion directions can be quantified (Basser et al. 1994). With DTI, water diffusion can be characterized by different diffusion parameters: (1) fractional anisotropy (FA) which refers to the selective directionality of diffusion in one direction compared to others (Beaulieu 2002). FA values range from 0 (isotropic diffusion, as in gray matter) to 1 (anisotropic diffusion, as in white matter), where higher values reflect faster diffusivity parallel to the fibers than perpendicular to them. Higher FA is an indicator of higher fiber density (Le Bihan et al. 2001), higher axonal organization (Alexander et al. 2007), or more myelinated fibers (Assaf and Pasternak 2008). (2) Mean diffusivity (MD), which measures the rotationally invariant overall magnitude of water diffusion; higher MD values indicate greater overall diffusion (Le Bihan et al. 2001). Calculations of FA and MD are based on extracting the radial diffusivity (RD; diffusion perpendicular to the axon) and axial diffusivity (AD; diffusion parallel to the axon), properties that provide more refined neurobiological information about white matter structure alteration (Alexander et al. 2007; Assaf and Pasternak 2008). DTI can delineate microstructural abnormalities affecting white matter pathways that may result in deficient function as determined by associated behavioral testing.

In this study we hypothesized differences in forebrain white matter integrity between children presenting with listening difficulties and atypical LEA, and typically developing (TD) children and typical REA in the dichotic competing words–free recall (CW-FR) subtest of the SCAN-3 test battery (Keith 2009). We predicted that, compared to the REA group, children in the LEA group would have lower FA values in frontal white matter, consistent with recent neuroimaging results (Schmithorst et al. 2013).

## Materials and Methods

### Participants

Twelve participants aged 7–14 years (mean 10.9 ± 2.1 years; 10 males) with auditory processing (AP) complaints were identified via chart review of the APD clinic at Cincinnati Children's Hospital Medical Center (CCHMC) in Cincinnati, OH. Children in this LEA group all had listening difficulties as reported by their parents. Complaints included difficulty understanding speech in the classroom and in noisy environments, difficulty following oral instructions, frequent requests to repeat oral information, and difficulty following directions despite normal hearing sensitivity. The performance of children in the LEA group was comparable to controls (see below) on several tests of auditory processing from the SCAN-3 battery (Keith 2009). However, they were identified by chart review as having an atypical LEA, and this was subsequently confirmed by further testing using the CW-FR subtest.

Twelve healthy TD children were recruited from the Cincinnati area via flyer and word of mouth. They were matched in age (7–14 years; mean 10.9 ± 2.25 years), sex (10 males), and handedness to the LEA group, but had typical REA on the CW-FR subtest.

All children, in both groups, were right-handed based on a questionnaire filled out by parents that included a question “Is your child right/left handed/inconsistent?” Parents were asked to respond based on which hand the child uses for writing, throwing, striking a match, scissors, toothbrush, spoon, knife, and a computer mouse. Only monolingual native English speakers with no known formal diagnosis of hearing loss, attention deficit disorder, or neurological impairment were included in the study. All experiments were conducted following the approval of the Institutional Review Boards at CCHMC and the University of Cincinnati. Each child filled an assent form and one parent filled an informed consent prior to starting the study.

### Audiological testing

Audiological testing was conducted in a sound-treated booth. Using a clinical audiometer, peripheral hearing sensitivity and the CW-FR subtest materials were delivered through insert phones. Pure tone thresholds from 250 to 8000 Hz were measured according to standard clinical procedures and were all <20 dB HL in both ears. Normal (A-type) middle ear status was verified through tympanometry for all participants. The CW-FR test was delivered from a compact disk according to the SCAN-3 manual and test instructions. Test materials consisted of monosyllabic word pairs, delivered dichotically at a level of 50 dB HL with two practice items to insure understanding of test procedure. Results were the number of correct words recalled from each ear, and the ear advantage (EA), calculated as the mathematical difference between right ear and left ear score, per the SCAN-3 manual. A positive EA number indicates REA and a negative EA number indicates LEA. Finally, EA scores were compared to age-normed criteria, per the SCAN-3 protocol, to determine whether scores fall within the typical or atypical range. All children in the LEA group had an atypical LEA (prevalence of 10% or less) compared to the normative data.

### DTI data acquisition and analysis

#### DTI scans

All scans were acquired on a Philips 3T Achieva system. Diffusion tensor echoplanar images (EPI) were acquired along 15 diffusion gradient directions for acquisition of 60 slices over the whole brain acquired for 2 mm isotropic resolution. The following parameters were used: TE = 62 msec, TR = 7600 msec, Gmax = 40 mT/m, slew rate = 200 T/m/sec, FOV = 22.4 × 22.4 cm, matrix = 112 × 112, slice thickness = 2 mm, value = 1000 sec/mm^2^, SENSE factor = 2. A 32-channel head coil and acquiring two signal averages for each acquisition was used to improve the signal to noise ratio (SNR).

#### DTI analysis

Preprocessing of the DTI scans was described in an earlier study (Schmithorst et al. 2013) where the same dataset was analyzed using machine learning techniques. In the current study, visual inspection of the scans was used to detect gross artifacts caused by nonideal RF and gradient performance or gross head motions causing misregestration. Ten of 34 datasets were discarded due to gross artifacts and head motion. Twenty-four datasets were included in the final analysis comprising 10 males and two females in each group. As the gender distribution was not balanced, gender and age were included as covariates in the analysis. Maps of FA, MD, axial diffusivity (AD), and RD were calculated from the diffusion-weighted images using Cincinnati Children's Hospital Image Processing Software (CCHIPS) incorporating routines written in the IDL software environment (ITT Visual Information Solutions, Boulder, CO). Spatial normalization to standard Montreal Neurological Institute (MNI) space was performed using routines written in SPM8 (Wellcome Institute of Cognitive Neurology, London, UK, RRID:nif-0000-00343) and whole-brain segmentation applied to the T1-weighted anatomical images was performed for each subject using procedures in SPM8. Using a six-parameter rigid-body transformation, the FA, MD, RD, and AD maps were coregistered to the white matter maps. Normalization of the white matter maps for each child to the white matter template was performed using the nonlinear normalization routine, and then applied to the DTI parameter maps. Only voxels with FA > 0.25 and white matter probability >0.9 were retained for further analysis with a minimum cluster size of 100 voxels. We only report clusters with a corrected *P*-value <0.01.

Data analysis was completed using the general linear model (GLM), with age and sex entered as covariates. Using FMRIB58_FA standard space template (FMRIB, University of Oxford, UK, RRID:nif-0000-00305), the group maps (FA, MD, RD, and AD) were projected onto the white matter skeleton in MNI space *Z*-score maps were generated and a 3-mm Gaussian filter was used with a threshold of *Z* = 8. Regions of interest (ROIs) were defined from clusters found to show a significant difference of DTI measures (FA, MD, RD, AD) between the groups. For each ROI, the centroid was computed, and then transformed from MNI coordinates to Talairach coordinates using the nonlinear mni2tal procedure outlined in http://www.nil.wustl.edu/labs/kevin/man/answers/mnispace.html (RRID:SciRes_000110).

Finally, the cortical gray matter region nearest to the centroid was found using the Talairach Daemon (Lancaster et al. 1997) and the appropriate white matter label was found using the MRI Atlas of Human White Matter (Oishi et al. 2010).

## Results

### Demographic and behavioral characteristics

Demographic and behavioral characteristics of the participants are shown in Table 1. There was no significant difference between the groups in age, sex, or the total score on the CW-FR subtest (*P* > 0.05). Based on the inclusion criteria, there was a significant difference between the groups in the number of words correctly identified in the right and left ear (*P* < 0.01; Table 1).

**Table 1:** Demographic and behavioral data on children having a left ear advantage (LEA; *n* = 12) or a right-ear advantage (REA; *n* = 12) on the competing words–free recall (CW-FR)

	REA	LEA	*P*
Group			
#Males, #females	10 M, 2 F	10 M, 2 F	1
Age (months) ±SD	131.1 ± 27.0	131.3 ± 25.0	0.98
CW-FR			
Total score ± SD	32.3 ± 2.3	31.5 ± 2.1	0.35
# Words correct in right ear	17.7 ± 1.1	12.0 ± 1.7	<0.001
# Words correct in left ear	14.1 ± 2.0	16.7 ± 2.3	<0.01

Data are means ± SD.

### DTI results

#### Group differences in fractional anisotropy

**Figure 1:**
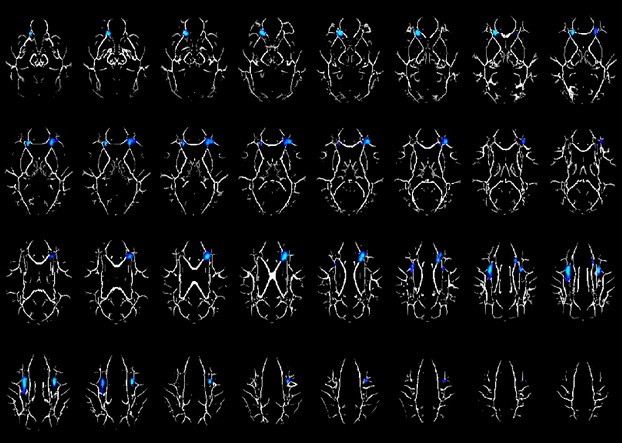
Regions with significant fractional anisotropy (FA). Difference between the left ear advantage (LEA) and the right ear advantage (REA) group (cold colors = LEA<REA) in a cohort of 24 children age 7–14 years old. Slice locations range from *z* = 18 to 49. All images are in radiological orientation.

Image analysis identified several distinct clusters showing decreased FA (*P* corrected <0.01) in the LEA compared to the REA children (see Table 2; Fig. 1). These clusters were located within frontal white matter regions. The regions' centroids were nearest to the right inferior and middle frontal gyrus (MFG; BA47 and BA10, respectively), left MFG (BA9 and BA10), left anterior cingulate (BA32), and frontal subgyral white matter bilaterally. While the nearest gray matter given by the Talairach Daemon for the right inferior and MFG was (BA47), visual inspection along with the MRI white matter atlas revealed that this white matter region was located in the genu of the corpus callosum.

**Table 2:** Group differences between the left ear advantage (LEA; *N* = 12) and the right ear advantage (REA; *N* = 12) in fractional anisotropy (FA)

Region	Contrast	*X*, *Y*, *Z* (MNI coordinates)	*X*, *Y*, *Z* (Talairach coordinates)	White matter label	Nearest gray matter (Brodmann's area)
Right frontal	LEA<REA	−18, 31, −2	−18, 30, −3	Genu of corpus callosum	Inferior frontal gyrus (BA47)
Right frontal	LEA<REA	−16, 31, −8	−16, 30, −8	Anterior corona radiata	Middle frontal gyrus (BA10)
Right frontal	LEA<REA	−28, 3, 32	−28, 4, 29	Superior corona radiata	Subgyral white matter
Left frontal	LEA<REA	30, 35, 14	30, 35, 11	Middle frontal gyrus WM	Middle frontal gyrus (BA10)
Left frontal	LEA<REA	22, 28, 26	22, 28, 23	Anterior corona radiata	Medial frontal gyrus (BA9)
Left frontal	LEA<REA	27, −3, 36	27, −1, 33	Superior corona radiata	Subgyral white matter
Left cingulate	LEA<REA	23, 28, 20	23, 28, 17	Anterior corona radiata	Anterior cingulate (BA32)

Coordinates are the centroid of the cluster and are reported in Montreal Neurological Institute (MNI) and Talairach stereotactic space. The nearest gray matter region and white matter labels are provided.

#### Group differences in mean diffusivity

**Figure 2:**
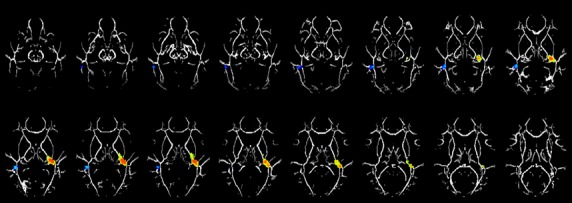
Regions with significant mean diffusivity (MD). Difference between the left ear advantage (LEA) and the right ear advantage (REA) group (hot colors =LEA>REA; cold colors = LEA < REA) in a cohort of 24 children age 7–14 years old. Slice locations range from *z* = 18 to 33. All images are in radiological orientation.

Mean diffusivity contrast analyses between the groups demonstrated two clusters with statistically significant group differences (*P* < 0.01, corrected). None of the clusters showing significant FA differences demonstrated any MD changes. However, the LEA group showed significantly increased MD in temporal white matter (the cluster centroid was closest to the transverse temporal gyrus [TTG], BA41) and decreased MD in temporal white matter (BA37; see Table 3; Fig. 2). Increased MD in TTG was accounted for by increase in both RD and AD. Again, visual inspection of this region along with the MRI white matter atlas revealed that this white matter region constituted the retrolenticular part of the internal capsule. However, the atlas does not distinguish between the retrolenticular and sublenticular parts.

**Table 3:** Group differences between the left ear advantage (LEA; *N* = 12) and the right-ear advantage (REA; *N* = 12) group in mean diffusivity (MD)

Region	Contrast	*X*, *Y*, *Z* (MNI coordinates)	*X*, *Y*, *Z* (Talairach coordinates)	White matter label	Nearest gray matter (Brodmann's area)
Left temporal	LEA>REA	32, −31, 8	32, −30, 9	Retrolenticular part of internal capsule	Transverse temporal gyrus (BA41)
Right temporal	LEA<REA	−50, −42, −10	−50, −41, −6	–	Subgyral (BA37)

Coordinates are the centroid of the cluster and are reported in Montreal Neurological Institute (MNI) and Talairach stereotactic space. The nearest gray matter region and white matter labels are provided.

#### Group differences in radial and axial diffusivity

**Figure 3:**
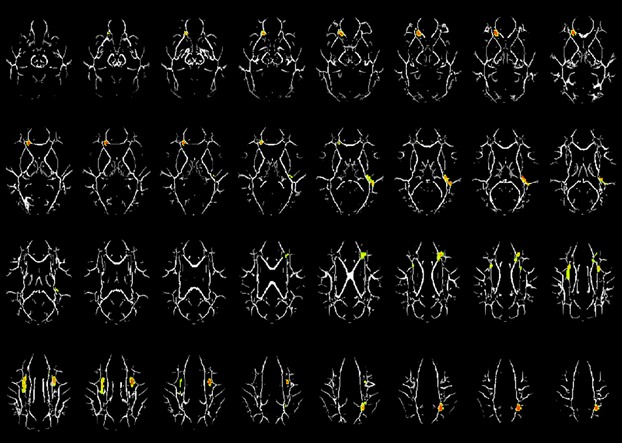
Regions with significant radial diffusivity (RD). Difference between the left ear advantage (LEA) and the right ear advantage (REA) group (hot colors = LEA>REA) in a cohort of 24 children age 7–14 years old. Slice locations range from *z* = 18 to 49. All images are in radiological orientation.

To further elucidate the white matter microstructure differences and the biological processes underlying LEA finding, RD and AD were examined in those regions exhibiting significant difference for FA or MD. Pairwise comparisons revealed a significant inverse pattern for group differences in RD and FA, where a significant decrease in FA in the LEA group was coupled with a significant increase in RD for nearly all clusters (Fig. 3; Table 4), except for the left MFG (BA10) and the left AC showing a significant decrease in FA, coupled with no change in RD but with significant increase in AD (see Tables 2005 and 2004).

**Table 4:** Group differences between the left ear advantage (LEA; *N* = 12) and the right ear advantage (REA; *N* = 12) group in radial diffusivity (RD)

Region	Contrast	*X*, *Y*, *Z* (MNI coordinates)	*X*, *Y*, *Z* (Talairach coordinates)	White matter label	Nearest gray matter (Brodmann's area)
Right frontal	LEA>REA	−18, 31, −1	−18, 30, −2	Genu of corpus callosum	Inferior frontal gyrus (BA47)
Right frontal	LEA>REA	−27, −3, 34	−27, −1, 31	Superior corona radiata	Subgyral white matter
Right frontal	LEA>REA	−14, 34, −8	−14, 33, −8	Anterior corona radiata	Middle frontal gyrus (BA10)
Left parietal	LEA>REA	23, −47, 44	23, −44, 43	Precuneus WM	Precuneus (BA7)
Left frontal	LEA>REA	22, 28, 28	22, 28, 24	Anterior corona radiata	Medial frontal gyrus (BA9)
Left temporal	LEA>REA	36, −36, 13	36, −34, 14	Retrolenticular part of IC	Transverse temporal gyrus (BA41)
Left cingulate	LEA>REA	21, 28, 22	21, 28, 19	Anterior corona radiata	Anterior cingulate (BA32)
Left frontal	LEA<REA	28, 1, 36	28, 3, 33	Superior corona radiate	Subgyral white matter

Coordinates are the centroid of the cluster and are reported in Montreal Neurological Institute (MNI) and Talairach stereotactic space. The nearest gray matter region and white matter labels are provided.

**Figure 4:**
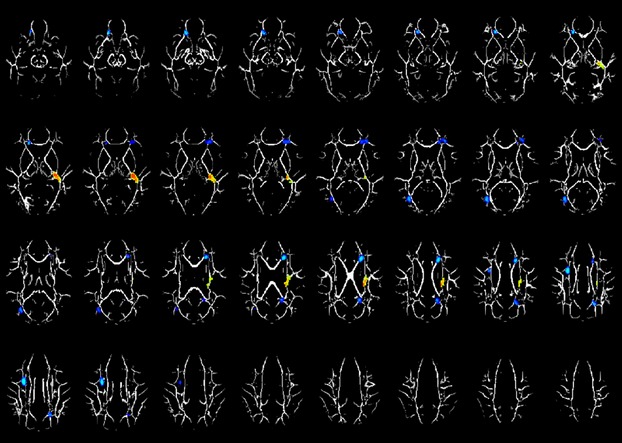
Regions with significant axial diffusivity (AD). Difference between the left ear advantage (LEA) and the right ear advantage (REA) groups (hot colors = LEA>REA; cold colors = LEA<REA) in a cohort of 24 children age 7–14 years old. Slice locations range from *z* = 18 to 49. All images are in radiological orientation.

As for AD, the LEA children showed significantly decreased AD in all regions showing significantly decreased FA (see Fig. 4; Table 5). Table 6 summarizes the relationship between the different measures of DTI investigated in this study.

**Table 5:** Group differences between the left ear advantage (LEA; *N* = 12) and the right ear advantage (REA; *N* = 12) group in axial diffusivity (AD)

Region	Contrast	*X*, *Y*, *Z* (MNI coordinates)	*X*, *Y*, *Z* (Talairach coordinates)	White matter label	Nearest gray matter (Brodmann's area)
Left sublobar	LEA>REA	28, −17, 24	28, −15, 23	Cortico spinal tract	Extranuclear white matter
Left temporal	LEA>REA	31, −31, 6	31, −30, 7	Retrolenticular part of internal capsule	Transverse temporal gyrus (BA41)
Right frontal	LEA<REA	−17, 32, −5	−17, 31, −6	Anterior corona radiata	Inferior frontal gyrus (BA47)
Right frontal	LEA<REA	−15, 32, −10	−15, 31, −10	Anterior corona radiata	Middle frontal gyrus (BA10)
Left frontal	LEA<REA	30, 35, 14	30, 35, 11	MFG WM	Middle frontal gyrus (BA10)
Left frontal	LEA<REA	21, 26, 24	21, 26, 21	Corpus callosum frontal	Medial frontal gyrus (BA9)
Left frontal	LEA<REA	28, 0, 36	28, 2, 33	Superior corona radiata	Subgyral white matter
Left cingulate	LEA<REA	21, 30, 24	21, 30, 21	Corpus callosum frontal	Anterior cingulate (BA32)
Right frontal	LEA<REA	−28, 2, 34	−28, 4, 31	Superior corona radiata	Subgyral white matter
Left cingulate	LEA<REA	22, −52, 24	22, −49, 25	Corpus callosum parieto-occipital	Cingulate gyrus (BA31)

Coordinates are the centroid of the cluster and are reported in Montreal Neurological Institute (MNI) and Talairach stereotactic space. The nearest gray matter region and white matter labels are provided.

**Table 6:** Summary of diffusion tensor imaging measures based on significant differences in FA: + = LEA>REA; − = LEA<REA

	FA	MD	RD	AD
Rt IFG- BA47	−		+	−
Rt MFG—BA10	−		+	−
Rt frontal subgyral	−		+	−
Lt MFG—BA10	−			−
Lt MFG—BA9	−		+	−
Lt ACC—BA32	−			−
Lt frontal subgyral	−		+	−
Lt retro/sublenticular part of IC		+	+	+

Rt, right; Lt, left; +, increase; −, decrease; FA, fractional anisotropy; MD, mean diffusivity; RD, radial diffusivity; REA, right ear advantage; AD, axial diffusivity; BA, Brodmann's area; MFG, middle frontal gyrus; IFG, inferior frontal gyrus; AC, anterior cingulate; IC, internal capsule.

## Discussion

Diffusion tensor imaging studies are very scarce in the APDs literature (Jerger 2004). This is the first study, to our knowledge, to investigate white matter microstructure differences in children presenting with listening difficulties. This study has shown white matter microstructural abnormalities in children with listening difficulties and an accompanying LEA compared to TD children with REA. The overall pattern of results suggests that first, disrupted connectivity to or from the frontal lobes, reflected by significantly lower FA, accounts for the major differences between the LEA and REA group. An increase in RD and a decrease in AD underlie the changes seen in FA. Second, significant increase in MD in the left retro/sublenticular part of the internal capsule was found in the LEA compared to the REA group. Increases in RD and in AD underlie the increase in MD, with no significant change in FA.

### Mechanisms underlying DTI abnormalities

Compared to the REA group, the LEA group demonstrated significantly lower FA in frontal multifocal white matter regions, adjacent to brain regions that have been implicated in attention and cognitive control function, including the prefrontal cortex, the left ACC and in frontal subgyral white matter. This finding is intriguing given the role of the prefrontal cortex and the ACC in higher order cognitive functions including directing attention (Lebedev et al. 2004; Wu et al. 2007), conflict and performance monitoring (Dosenbach et al. 2006), response inhibition, and error detection (MacDonald et al. 2000). Furthermore, the finding of decreased FA in the genu of the corpus callosum (CC) is also important, given the major role of the CC in interhemispheric communication and, in particular, in dichotic listening performance (see Westerhausen and Hugdahl 2008 for a review). However, the genu part of the CC is known to interconnect frontal cortical regions in the two hemispheres (Yazgan et al. 1995). Abnormality in this area (genu) is thus postulated to affect frontal networks associated with cognitive function and may therefore play a role in top–down management problems in auditory processing in the LEA group. In addition, integrity of the CC is critical for better left ear recall, and the LEA group demonstrated better and improved recall from the left ear, hence it was not expected to find abnormal callosal connection in posterior parts of CC connecting temporal lobes.

Additional important finding in the current study was increased MD in the left sublenticular part of the internal capsule (IC). The sublenticular part contains corticothalamic and thalamocortical fibers (auditory radiations) connecting to the auditory cortex. Disruption in fibers connecting the dominant contralateral pathway, from the right ear to the left auditory cortex, suggests that the right ear input may experience less efficient processing. The structural abnormality reported here in the left sublenticular part of internal capsule (auditory radiations) may contribute to the inferiority of right ear compared to left ear recall during dichotic listening, as proposed by the “structural model” (Kimura 1963a,b). Future studies should use HARDI fiber tracking (Berman et al. 2013) to delineate the exact extent of the auditory radiations which cannot be reliably defined using voxel-based DTI analysis or DTI fiber tracking (Behrens et al. 2007).

Neuropathology affecting white matter fibers often causes decreased FA, indicative of scattered, unhealthy or poorly myelinated white matter fibers (Beaulieu 2002) possibly triggering connectivity and neural communication disruptions between brain areas. However, decreased anisotropy may not provide sufficient information to depict specific tissue changes as it may result from different combination changes in RD and AD (Alexander et al. 2007). Hence, in the current study, we assessed changes in other diffusion parameters such as AD and RD, in regions demonstrating significant between-groups FA and MD differences (see Table 6).

### Fractional anisotropy

One novel result of this study was decreased FA in multifocal frontal white matter, which was largely accounted for by significantly increased RD and decreased AD among children in the LEA group. In view of the fact that RD reflects restricted diffusion perpendicular to the axonal pathway due to myelin bundles (Alexander et al. 2007), increased RD in our study suggests reduced or delayed myelin development in the LEA group compared to the REA group. This finding is consistent with previous investigations demonstrating increased RD in a mouse model of dysmyelination (Song et al. 2002); while other studies reported increased RD with decreased AD in shiverer mouse model with dysmyelination (Harsan et al. 2006; Tyszka et al. 2006).

In addition, decreased AD was further found in the LEA group in all clusters displaying significant FA decrease. While future research is needed to elucidate the biological correlates of AD, several factors have been proposed to cause changes in AD. Those include decreased fiber coherence and organization (Dubois et al. 2008), growth of neurofibrils and glial cells during brain development leading to increased tortuosity of the extra-axonal space, axonal pruning reducing overabundant axons (Bockhorst et al. 2008), or axonal injury (Kim et al. 2006; Budde et al. 2009). However, decreased AD observed in our study coupled with increased RD with no significant changes in MD is most consistent with decreased fiber organization and decreased myelination (Alexander et al. 2007; Dubois et al. 2008).

As is true for many neural circuits in the brain, central auditory circuits rely on accurate, fast, and dependable neurotransmission to process auditory information (Kim et al. 2013). Myelin is critical for high-speed and accurate conduction of electrical impulses through axons and controls the synchrony of impulse transmission between spatially distant cortical regions deemed critical for perception and cognitive function. Deficits in myelin insulation can disrupt the accuracy needed (millisecond precision) for the coincident arrival and firing of synaptic signals (Fields 2008) and consequently, may lead to sensory and cognitive deficits. It is possible that the microstructural abnormalities in the LEA group, suggesting decreased myelination in pathways connecting frontal regions, may cause slowed or desynchronized impulse conduction in cortical networks resulting in impairment in tasks integration necessary for listening and cognitive function. From this perspective, listening difficulties in the LEA group may involve inability to integrate a collection of separate processing features despite a preserved ability to process individual features (Frith 1989), providing an explanation for normal tone sensitivity but impaired listening.

Alternatively, the results suggest that altered connectivity in the LEA group may indicate disrupted myelination and/or alterations in axonal architecture associated with delayed maturation. Several lines of evidence pointed to delayed maturation of some white matter pathways, especially pathways connecting to and from PFC (Paus 2005; Lebel et al. 2008). Nevertheless, significant differences between the groups in the current study can provide an early biomarker of disrupted connectivity as reflected by the difference between neuromaturation related to age and pathology. This observation of both increased RD and decreased AD suggests that differences in structural connectivity might be guided by more than one underlying mechanism.

### Mean diffusivity

Increased MD in the left sublenticular part of the internal capsule (auditory radiations) was seen in the LEA compared to the REA group and was derived from increase in both RD and AD. MD is known to decrease with age, however, the precise cause for this decrease is not established. It is thought to be due to the simultaneous decrease in overall water content and the proliferation and maturation of glial cell bodies leading to increase in membrane density (Neil et al. 2002; Dubois et al. 2008). The increase in MD (increased diffusivity in all directions) with concomitant increase in RD in our study suggests late maturational processes in the region where auditory input is transmitted between the thalamus and the auditory cortex. Again, auditory thalamocortical radiations are known to mature later than the visual thalamocortical radiations, however, adult level of myelin is achieved around age 4 years (Moore and Guan 2001). Consequently, late maturational processes in the LEA, but not the REA group can provide an early biomarker of pathology.

### Structural neuropathy underlying other neurodevelopmental disorders in children

Studies of other neurodevelopmental disorders (e.g., specific language impairment, attention deficit disorder, autism spectrum disorder) that are highly comorbid with- or possibly indistinguishable from APD (Sharma et al. 2009; Ferguson et al. 2011), also show impaired white matter microstructure in frontal networks.

In the ADHD literature, the effects of structural abnormalities of frontal white matter on function have been investigated extensively (Ashtari et al. 2005; Casey et al. 2007; Konrad et al. 2010). Several lines of evidence support the hypothesis that altered structural connectivity, in frontostriatal pathway and specifically in PFC white matter, might contribute directly to the pathophysiology of ADHD (see Liston et al. 2011 for a review).

A preliminary study by Ashtari et al. (2005) demonstrated decreased FA, predominantly in frontal and cerebellar white matter, in children with ADHD. Another study of adults with childhood ADHD reported decreased FA in the right cingulum and in the right superior longitudinal fasciculus (Makris et al. 2008). Based on evidence that those bundles are parts of the attention and executive control system, the authors concluded that they are involved in the pathophysiology of ADHD. Casey et al. (2007) showed FA in prefrontal white matter to correlate with measures of impulsivity in child–parent ADHD. Finally, structural MRI studies in children with ADHD reported reductions in anterior CC which correlated significantly with impulsivity and hyperactivity symptoms (Hynd et al. 1991; Giedd et al. 1994). Collectively, there is convergent evidence that disruption in frontal/prefrontal white matter circuitry, and in the anterior CC, may be related to neurobiological deficits underlying inattention and cognitive control. Behaviorally, Sutcliffe et al. (2006) demonstrated the effect of attention state on auditory processing abilities in children with ADHD, on and off medication. Their results suggest modulation of auditory processing abilities by the frontal lobe circuitry.

In the literature of attentive listening and auditory attention to speech processing, similar networks involving the frontal lobe have been implicated. These networks include a fronto-parietal attention network and a medial-lateral frontal cognitive control network consisting mainly of the mid-PFC, ACC, and inferior parietal areas, as well as the anterior insula and precentral gyrus (Shaywitz et al. 2001; Fritz et al. 2007; Christensen et al. 2008; Westerhausen et al. 2010). Thus, from this perspective, it is not unexpected that alterations (reflected by decreased FA) in white matter connecting nodes of this network, as seen in the current study, will have functional relationships in the LEA group.

In the autistic spectrum disorders (ASD) literature, studies have shown decreased FA and increased MD in multiple white matter tracts but most consistently in frontal regions, corpus callosum, cingulum, and aspects of the temporal lobe (Bloemen et al. 2010; Shukla et al. 2010; also see Travers et al. 2012 for a review). Decreased FA was often accompanied by increased RD, similar to our current results. Similar results are reported in the developmental dyslexia literature where DTI studies generally show correlation between lower FA values in left frontal and temporoparietal areas and poor reading ability or dyslexia (for a review, see Vandermosten et al. 2012).

Our results thus agree with previous findings in children with other neurodevelopmental disorders (e.g., ADHD, ASD) demonstrating decreased FA accompanied by increased RD in pathways connecting to and from the frontal lobe (Barnea-Goraly et al. 2004; Nagel et al. 2011; Lawrence et al. 2013). Furthermore, our results argue that frontal white matter and brain connectivity may be impacted in children with listening difficulties. Affected frontal regions encompass critical nodes in the fronto-parietal attention network, the medial-lateral frontal cognitive control network, and the fronto-striatal network. This provides further evidence that auditory processing problems, particularly in populations with atypical LEA, may have their roots in the top–down attentional networks that modulate auditory attention and processing. This finding also supports one of the major hypotheses concerning APD; namely that APD stems from a deficient top–down cognitive function, arising from multimodal processing centers in the brain (Moore et al. 2010) and that listening difficulties and APD may reflect a more general “neurodevelopmental syndrome” (Moore and Hunter 2013).

Interestingly, a recent study investigating white matter microstructure in children with sensory processing disorder (SPD), including auditory dysfunction, reported reduced white matter integrity predominantly in posterior cerebral tracts (Owen et al. 2013). Although there is a discrepancy between our results and their findings in location of disrupted white matter, DTI measures demonstrated a similar trend. Namely, decreased FA, increased MD, and RD compared to TD children. Possible reasons for the discrepancy with respect to the results of the two studies include the sample heterogeneity, using a parent questionnaire to assess sensory ability, and the comorbidity evident in the Owen's study.

In summary, our results and recent neuroimaging findings indicate that the etiology of listening difficulties and a LEA finding for speech-related stimuli in dichotic listening is not purely sensory and that higher order deficits, specifically attention, might play a vital role in explaining this finding. Specifically, multifocal white matter disruptions, reflected by decreased FA, in the LEA group were identified in regions important for executive function, attention, and response inhibition; with most consistent findings in regions involving the ventral and dorsal prefrontal cortex and the dorsal ACC white matter. Disruption within these nodes or in the connectivity between them might provoke disruption in the network as a whole providing a biomarker for listening difficulties in this population.

## Limitations and Future Research

Our preliminary study is subject to some limitations. First, the control group (REA group) consisted of TD children with typical REA who did not present with listening difficulties. Additionally, a group of TD children with REA referred for APD testing due to listening difficulties would have augmented the ability of this study to delineate the relative significance of LEA finding in children with auditory processing deficits.

Second, participants in both groups were strongly right-handed based on questionnaires filled by parents. However, handedness and hemispheric language dominance do not go hand in hand (Szaflarski et al. 2012) and there was no direct measure for hemispheric language dominance. Finally, measures of language skills and overall cognitive function were not controlled for.

This preliminary study is the first, to our knowledge, to investigate white matter microstructure in children with atypical LEA and listening difficulties. Future studies in the field are needed to delineate microstructural abnormalities in the APD population/subgroups employing hypothesis-driven methodology (e.g., tractography; Behrens et al. 2003) to establish structure–function association between specific axonal pathways and listening difficulties/APD. Behavioral measures, both sensory and supramodal might provide critical correlates to the structural signature and together may constitute more sensitive means for diagnosing APD.

## Conclusions

Our results suggest that LiD/APD represent a disorder of altered structural connectivity of the brain, revealed by frontal distributed atypical white matter microstructure. Furthermore, results suggest delayed myelination in frontal multifocal white matter regions and in the region of auditory radiations (auditory input is transmitted between the thalamus and the auditory cortex). Together, our findings reveal that both sensory and supramodal deficits may underlie the differences between the groups and may pinpoint biomarkers of listening difficulties in children.
